# *De Novo* Genome Assembly of the Meadow Brown Butterfly, *Maniola jurtina*

**DOI:** 10.1534/g3.120.401071

**Published:** 2020-03-11

**Authors:** Kumar Saurabh Singh, David J. Hosken, Nina Wedell, Richard ffrench-Constant, Chris Bass, Simon Baxter, Konrad Paszkiewicz, Manmohan D Sharma

**Affiliations:** * College of Life and Environmental Sciences, University of Exeter, Penryn, UK; † School of Biological Sciences, University of Adelaide, Adelaide, Australia; ‡ College of Life and Environmental Sciences, University of Exeter, Exeter, UK

**Keywords:** Genome assembly, Lepidoptera, *comparative genomics*, *Maniola jurtina*, meadow brown

## Abstract

Meadow brown butterflies (*Maniola jurtina*) on the Isles of Scilly represent an ideal model in which to dissect the links between genotype, phenotype and long-term patterns of selection in the wild - a largely unfulfilled but fundamental aim of modern biology. To meet this aim, a clear description of genotype is required. Here we present the draft genome sequence of *M. jurtina* to serve as a founding genetic resource for this species. Seven libraries were constructed using pooled DNA from five wild caught spotted females and sequenced using Illumina, PacBio RSII and MinION technology. A novel hybrid assembly approach was employed to generate a final assembly with an N50 of 214 kb (longest scaffold 2.9 Mb). The sequence assembly described here predicts a gene count of 36,294 and includes variants and gene duplicates from five genotypes. Core BUSCO (Benchmarking Universal Single-Copy Orthologs) gene sets of Arthropoda and Insecta recovered 90.5% and 88.7% complete and single-copy genes respectively. Comparisons with 17 other Lepidopteran species placed 86.5% of the assembled genes in orthogroups. Our results provide the first high-quality draft genome and annotation of the butterfly *M. jurtina*.

The meadow brown butterfly (*Maniola jurtina*, NCBI:txid191418) is a member of the nymphalid subtribe Satyrini. It is an important model organism for the study of lepidopteran ecology and evolution and has been extensively studied by ecological geneticists for many years (Dowdeswell *et al.* 1949; Dowdeswell 1961; Ford 1965). Found across the Palearctic realm it primarily habituates in grasslands, woodland rides, field-margins and can even be found in overgrown gardens.

The species displays marked sexual dimorphism. Females are more colorful than males and have large upper-wing eyespots (Figure 1). It also exhibits considerable quantitative variation in the sub-marginal spot pattern of its wings (Brakefield and van Noordwijk 1985) and therefore represents an ideal model in which to dissect the links between genotype, phenotype and long-term patterns of selection in the wild (Baxter *et al.* 2017) - a largely unfulfilled but fundamental aim of modern biology. This draft genome and corresponding annotations will offer a core resource for ongoing work in lepidopterans and other arthropods of ecological importance.

**Figure 1:**
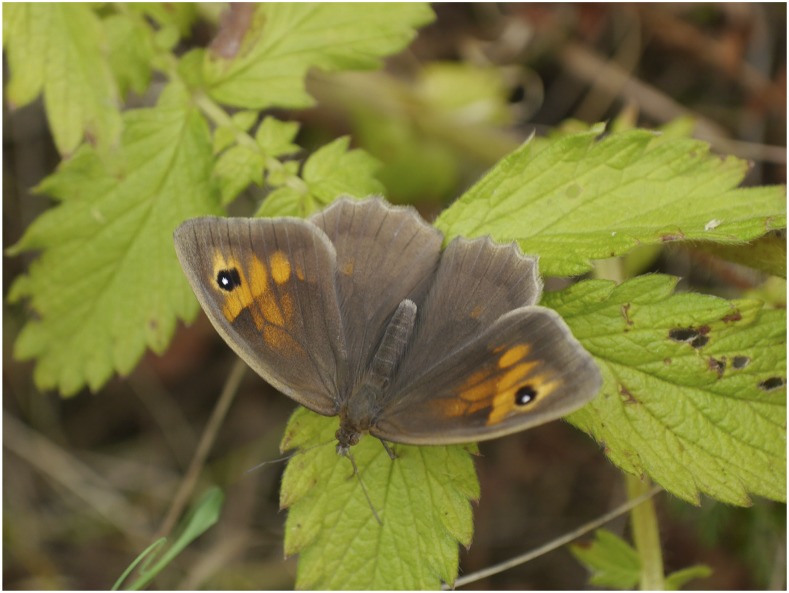
Female *Maniola jurtina* (picture credit: Richard ffrench-Constant).

## Materials and Methods

### Sampling and sequencing

Adult meadow brown (*Maniola jurtina*) butterflies were collected from multiple fields (Isles of Scilly, Cornwall) in June 2012, anesthetized by refrigeration for 2 hr and then killed by subsequent freezing. High molecular weight genomic DNA was isolated from whole body (pooled, excluding wings) of five individual females using the genomic-tip 100/G kit (Qiagen, Hilden, Germany) supplemented with RNase A (Qiagen, Hilden, Germany) and Proteinase K (New England Biolabs, Hitchin, UK) treatment, as per the manufacturer’s instructions. DNA quantity and quality were subsequently assessed using a NanoDrop-2000 (Thermo Scientific, Loughborough, UK) and a Qubit 2.0 fluorometer (Life Technologies). Molecular integrity was confirmed using pulse-field gel electrophoresis.

Illumina data (100bp paired-end) was generated using standard Illumina protocols for a 250-500 bp PE library and multiple mate-pair libraries ranging between 180 to 7k bp (Table S1). 20 kb PacBio libraries were generated and size-selected following the manufacturers recommended protocols and sequenced on 18 SMRT cells of the RSII instrument. Finally, long reads (longest read 300Kb) were obtained using the Oxford Nanopore Technologies MinION platform (R7.4) (Table S2). Illumina, PacBio and MinION library preparation and sequencing were performed by the Exeter Sequencing Service, University of Exeter.

### Genome assembly

The genome characteristics of *M. jurtina* were estimated using a k-mer based approach implemented in GenomeScope (Vurture *et al.* 2017). Short-read Illumina reads were quality filtered and subjected to 19-mer frequency distribution analysis using Jellyfish -v2.2.0 (Marçais and Kingsford 2011).

**Figure 2:**
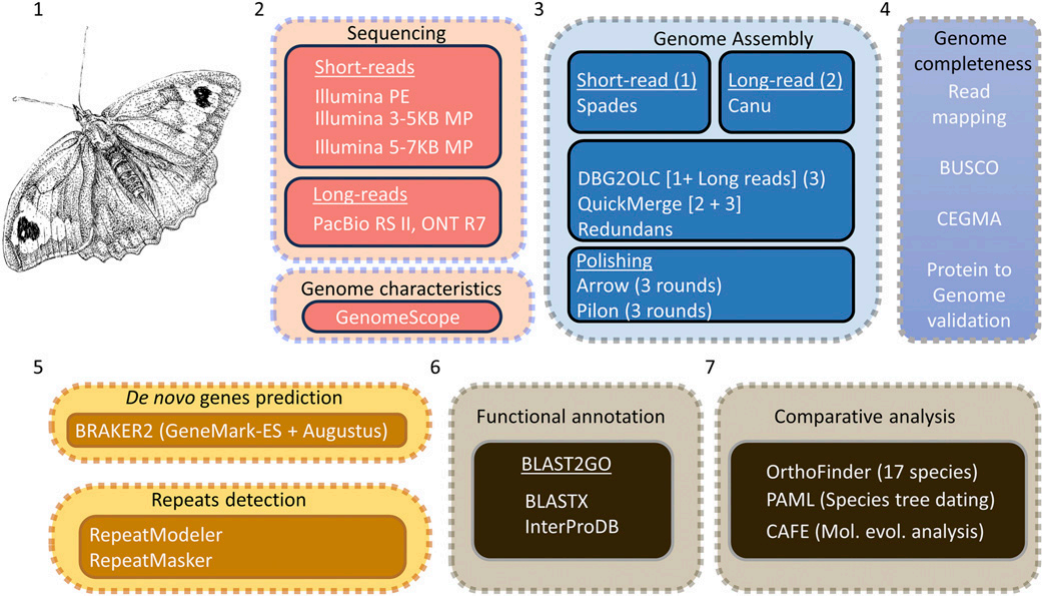
Schematic overview of the workflow used for sequencing, genome size estimation, assembly and annotation of the *M. jurtina* genome. 1. An artist’s impression of a female *M. jurtina* (samples collected from multiple fields and processed for DNA extraction); 2. Multiple sequencing approaches adopted along with genome characterization using genome scope; 3. Genome assembly using a hybrid approach; 4. Genome completeness assessment; 5. *De novo* genes prediction and repeat detection; 6. Functional annotation; 7. Comparative analysis. Note that transcriptome data (orange segment) were obtained from publicly available sources at NCBI and only used for genome annotation.

Genome assembly was performed by adopting a novel hybrid approach (Figure 2). Paired-end Illumina reads were trimmed and filtered for quality values using Trim Galore -v0.4.2 (Krueger 2016) and assembled using Spades -v3.9.1 (Bankevich *et al.* 2012). Long reads obtained from MinION were mixed with PacBio reads and assembled using Canu -v1.3.0 (Koren *et al.* 2017). The short-read assembly was further assembled along with long-reads using DBG2OLC -v20160205 (Ye *et al.* 2016). Canu and DBG2OLC assemblies were later merged using QuickMerge -v0.3.0 (Chakraborty *et al.* 2016) and redundancy reduction, scaffolding and gap closing were carried using Redundans -v0.14c (Pryszcz and Gabaldon 2016). The draft assembly was polished using arrow (part of the genomicconsensus package in PacBio tools) -v2.3.2 (Pacific Biosciences of California), which exclusively mapped long PacBio reads against the draft assembly using the BLASR pipeline (Chaisson and Tesler 2012). The draft assembly was also polished with the Illumina short-reads using Pilon -v1.23.0 (Walker *et al.* 2014).

### Evaluation of the completeness of the genome assembly

The completeness of the draft genome was assessed by mapping raw short and long reads against the assembly. BUSCO (Benchmarking universal single-copy orthologs) -v3.0.2 (Simão *et al.* 2015) and CEGMA (Core Eukaryotic genes mapping approach) -v2.5.0 (Parra *et al.* 2007) were used to check genomic completeness of the assembly. In the case of BUSCO, Arthropoda and Insecta gene sets were compared against the assembly. We also assessed the completeness of this assembly by aligning complete genomes of *M. jurtina* genome against *H. melpomene* and *B. anynana* (a close relative) using Mummer -v3.1.0 (Kurtz *et al.* 2004).

### Genome annotation

Before predicting gene models, the genome of *M. jurtina* was masked for repetitive elements using RepeatMasker -v4.0.7 (Smit 2013–2015). RepeatModeler -v1.0.11 (Smit 2008–2015) was used to model the repeat motifs and transposable elements. Repeats originating from coding regions were removed by performing a BLAST search against the *B. anynana* proteins. Sequence with hits at E-value > 1e^-10^ were filtered out. The RepBase *-v*24.05 library was then merged with the repeats predicted by RepeatModeler and used to mask the *M. jurtina* genome. Protein coding genes were predicted using GeneMark-ES -v4.3.8 (Lomsadze *et al.* 2005) and AUGUSTUS -v3.3.0 (Stanke and Morgenstern 2005) implemented in the BRAKER -v 2.1.2 (Hoff *et al.* 2016) pipeline using species-specific RNA-seq alignments as evidence. Publicly available *M. jurtina* RNA-seq datasets (SRR3724201, SRR3724266, SRR3724269, SRR3724271, SRR3724198, SRR3724196, SRR3724195, SRR3721773, SRR3721752, SRR3721684, SRR3721695) were downloaded from NCBI and mapped individually against the repeat masked genome using STAR -v2.7.1 (Dobin *et al.* 2013). The bam files from individual samples were then combined using custom scripts and then fed into BRAKER. Functional annotation of the *de-novo* predicted gene models was carried out using homology searches against the NCBI nr database and Interpro database using BLAST2GO -v5.2.5 (Gotz *et al.* 2008).

### Comparison to other Lepidopteran species

To characterize orthology and investigate gene family evolution across Lepidoptera, the final annotation set of *M. jurtina* was compared to 17 other genomes including a dipteran (*Drosophila melanogaster*), and a trichopteran (*Limnephilus lunatus*) as outgroups. The proteomes of *Amyelois transitella* v1.0, *B. anynana* v1.2, *Bombyx mori* v1.0, *Calycopis cecrops* v1.1, *Chilo suppressalis* v1.0, *Danaus plexippus* v3.0, *Heliconius melpomene* v2.0, *Junonia coenia* v1.0, *Limnephilus lunatus* v1.0, *Melitaea cinxia*, *Operophtera brumata* v1.0, *Papilio polytes* v1.0, *Phoebis sennae* v1.1, *Plodia interpunctella* v1.0, *Plutella xylostella* v1.0 were downloaded from Lepbase. OrthoFinder -v1.1.8 (Emms and Kelly 2018 *preprint*) was used to define orthologous groups (gene families) of genes between these peptide sets.

### Phylogenetic tree construction and divergence time estimation

Phylogenetic analysis was performed using 39 single-copy orthologous genes, conserved among 17 species, using OrthoFinder. Additionally, OrthoFinder generated a species tree where *D. melanogaster* was used as the outgroup. The species tree was rooted using the STRIDE -v1.0.0 (Emms and Kelly 2017) algorithm implemented in OrthoFinder. MCMCTREE, as implemented in PAML -v4.9e (Yang 2007), was then used to estimate the divergence times of *M. jurtina* with approximate likelihood calculation. For this, the substitution rate was estimated using *codeml* by applying root divergence age between the Diptera, Lepidoptera and Trichoptera as 350 MY (Kjer *et al.* 2015). This is a simple fossil calibration of 350 MY for the root. The estimated substitution rate was the per site substitution rate for the amino acid dataset and used to set priors for the mean substitution rate in Bayesian analysis. As a second step, the gradient (g) and Hessian (H) of branch lengths for all 17 species were also estimated. Finally, the tree file with fossil calibrations, the gradient vector and hessian matrices file and the concatenated genes alignment information were used in the approximate likelihood calculation. The parameter settings of MCMCTREE were as follows: clock = 2, model = 3, BDparas = 110, kappa_gamma = 6 2, alpha_gamma = 11, rgene_gamma = 9.09, and sigma2_gamma = 1 4.5. Finally, Gene family evolution across arthropods was investigated using CAFE -v3.0 (De Bie *et al.* 2006). Scripts used for the analysis of genomic data are available at: https://github.com/kumarsaurabh20/Maniola_jurtina_genome_sequencing

### Analysis of spot pattern related genes

To test whether any genes involved in wing or spot -pattern formation across Lepidoptera were identifiable in the current *Maniola* assembly, we first performed a wide literature search on PubMed (https://www.ncbi.nlm.nih.gov/pubmed/) using the keywords *Lepidoptera*, *butterfly*, *wing*, *spot*, *pattern*, *gene* and then manually filtered through the results to generate a list of candidate genes (Table 1 and Table 3).

**Table 1:** *jurtina* genome properties

Properties	Genome
# scaffolds (> 1000 bp)	10,860
Total length (>= 1000 bp)	618,415,580
Largest scaffold	2,944,739
Total length	618,415,580
GC (%)	36.90
N50	214,423
N75	78,459
L50	658
L75	1,875
# N’s per 100 kbp	8,864.86

This includes a selection of regulators possibly responsible for pattern variation (*APC*, *Naked cuticle*), transcription factors linked with eyespot patterning (*Distal-less*, *Dll*, and *Engrailed*, *En*), along with other transcription regulators such as *Apterous* and *DP*. Additionally, we considered *poik* (HM00025), also known as *cortex*, *Optix*, *Doublesex*, *Hox*, *Vermilion* and *black* (pigment synthesis) along with the *Ecdysone receptor* (*EcR*) involved in wing pattern plasticity.

NCBI esearch and efetch tools were used to filter (*NOT partial NOT hypothetical NOT uncharacterized*), and query individual spot pattern proteins across Lepidoptera using both, full and abbreviated protein names where available (Table 3; total 1347 homologs) and then these proteins were queried against the *Maniola* genome using Exonerate -v2.2.0 (Slater and Birney 2005) protein2genome model with the following customised options *–refine region–score 900–percent 70 -S FALSE –softmasktarget TRUE –bestn 1–ryo \”>%ti (%tab - %tae) coding (%tcb - %tce) cds_length (%tcl)\n%tcs\n”*.

### Data availability

The raw sequencing data and genome assembly have been deposited at the NCBI SRA database under the BioProject PRJNA498046 and genome accession number VMKL00000000. Blast results, annotation and proteome associated with this manuscript are available at https://zenodo.org/record/3352197. Scripts used for the analysis of genomic data are available at: https://github.com/kumarsaurabh20/Maniola_jurtina_genome_sequencing. Supplemental material available at figshare: https://doi.org/10.25387/g3.11594187.

## Results and Discussion

### Genome assembly

Sequencing of short-read libraries, both paired-end and mate-pairs, produced 317.1 million read pairs with an average insert size of 524.8 bp. Analysis of the unimodal 19-mer histogram with a coverage peak at 17x suggested an expected genome size of 576 MB (see Materials and Methods). Note here, that although the genome size estimated via this method is strongly dependent on the sequencing read-depth, based on the genome size of the most closely related species *Bicyclus anynana* (475 Mb), this estimate does not seem inordinate. The estimated heterozygosity rate was in the range of 1.89–1.93% (Table S3) and the genome was comprised of approximately 76% repetitive elements that are likely to contain units of highly repetitive W chromosome as the samples used in this study were all female (Table S3). We next performed a *de novo* genome assembly using a hybrid approach (see Materials and Methods). Spades assembly using multiple k-mer values produced 53,043 scaffolds having a total length of 319.9 Mb and N50 of 48.073 Kb. Long-read library sequencing produced 18.08 Gb (total 2398917 reads greater than 1000 bp) of data giving 21.7x overall sequencing coverage (Table S2). Canu assembled 10,463 contigs with N50 of 32.9 Kb. To further improve genome contiguity, we used DBG2OLC which is based on a hybrid approach of using both long- and short-reads. This assembly resulted in 46,361 short-read polished contigs with N50 of 60.26 kb which is an improvement of 12Kb over Spades assembly. In view of the recent developments in the hybrid assemblers, we further explore combining DBG2OLC assembly with long-read only Canu assembly using Quickmerge, an approach known to achieve high genomic contiguity with modest long- and short-read sequencing coverage. Merging of two assemblies with Quickmerge produced 30,457 contigs with a further improved N50 of 92.57 Kb. The assembly size, however, in Quickmerge step (762.9 Mb) surpassed the expected genome size of 576 Mb. To remove the alternate haplotypes from the assembly and reduce the inflated genome size, we added a redundancy removal step by using Redundans. This step improved the N50 by removing haplotypes and reducing the total assembly size. The final genome assembly comprised 618 Mb with 36.9 GC% and N50 of 214Kb (Table 2). Detailed assembly properties are given in Table 1 and Table S4.

**Table 2:** Different assembly versions, data, software used and summary statistics

Version	Data	Assembler	N50	#Sequences	Total length
1	Short-read PE	Spades	48,073	53,043	319,930,151
2	Long-reads (PacBio + MinION)	Canu	32,954	10,463	296,564,618
3	Version 1 + Long-read (PacBio + Minion)	DBG2OLC	60,269	46,361	317,966,984
4	Version 2 + 3	QuickMerge	92,579	30,457	762,970,634
5	Version 4 + PE + MP + PacBio + MinION	Redundans	213,669	10,863	616,464,047

### Evaluation of the completeness of the genome assembly

To evaluate the completeness of the genome assembly, we first mapped raw short and long reads against it. The percentage of aligned reads ranged from 94 to 95% using paired-end and mate-paired short reads. Then we assessed the gene completeness using BUSCO and CEGMA. About 90.5% and 88.7% total BUSCO genes were identified in the Arthropoda and Insecta sets respectively. Additionally, 91% CEGMA genes, both complete and partials, were successfully found in the assembly (Table S5 and S6). The number of matches found between *M. jurtina* and *B. anynana*, after whole genome alignment, were significantly more as compared to *H. melpomene*. The genome size of *H. melpomene* (∼250 MB) is smaller than *B. anynana* (∼475 MB). Therefore many *M. jurtina* genomic sequences ended up with no hits.

### Genome annotation

Annotation of the *M. jurtina* genome was carried out using the BRAKER pipeline. 11 publicly available datasets (See Material and Methods) were downloaded from NCBI totalling 116.4 million single-end transcriptomic reads. To predict genes, the reads were aligned against the *M. jurtina* assembly. BRAKER pipeline resulted in 38,101 genes after removing low quality genes with fewer than 50 amino-acid and/or exhibiting premature termination. In the final gene set, mean gene length, mean CDS length, mean intron length and exon number per gene were 4,144 bp, 976 bp, 921 bp and 5 respectively (Table S7). Approximately 34,263 out of 38,101 genes (90%) of the predicted genes could be assigned functional annotation based on BLAST searches against the non-redundant protein database of NCBI and InterPro.

### Comparison to other Lepidopteran species

**Figure 3:**
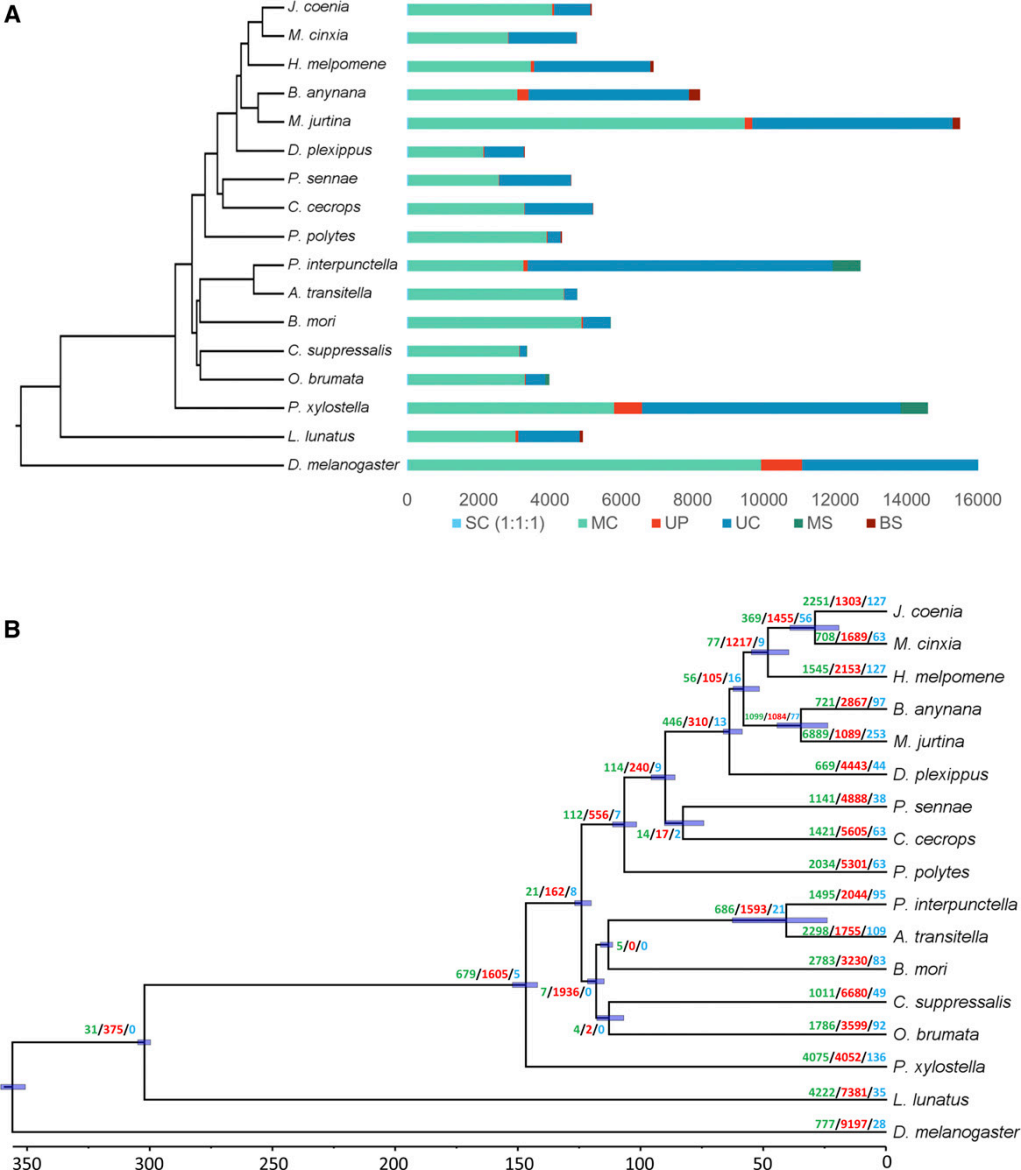
Evolutionary and comparative genomic analysis. (A) Ortholog analysis of *M. jurtina* with 16 other arthropod species. SC indicates common orthologs with the same number of copies in different species, MC indicates common orthologs with different copy numbers in different species, UP indicates species specific paralogs, UC indicates all genes which were not assigned to a gene family, MS indicates moths specific genes and BS indicates butterfly specific genes. (B) Species phylogenetic tree and gene family evolution. Numbers on the node indicate counts of the gene families that are expanding (green), contracting (red) and rapidly evolving (blue).

For comparative genomics analysis, we analyzed the orthologous gene relationships among several species (see Materials and Methods and Table S8). The combined gene count of these species was 349,442 of which 86.5% were assigned to 15,064 orthogroups. 50% of all genes were in orthogroups with 23 or more genes and were contained in the largest 4439 orthogroups. There were 2915 orthogroups with all species present and 39 of these consisted entirely of single-copy genes. A total of 216 gene families were specific to *M. jurtina* compared to 627 and 1716 in butterfly and moths respectively (Figure 3A).

### Phylogenetic tree construction and divergence time estimation

**Figure 4:**
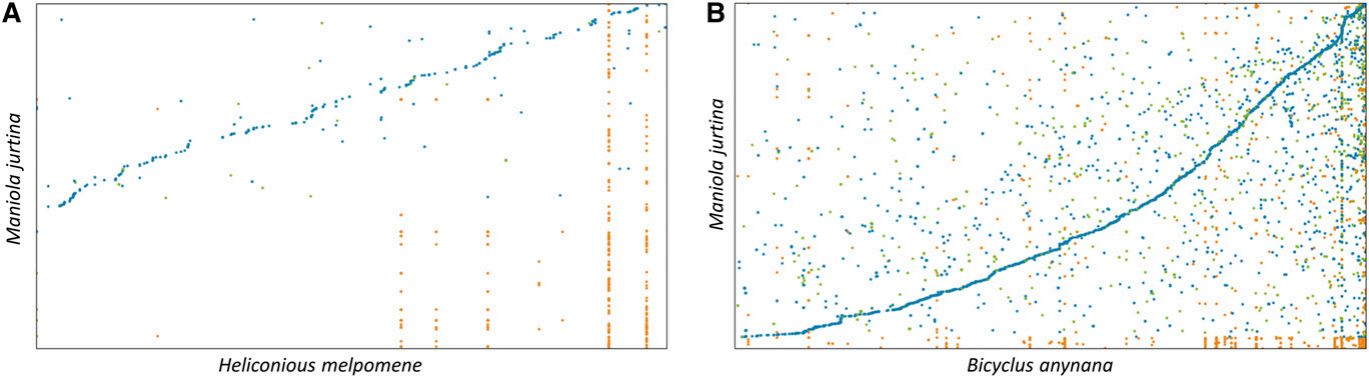
Genome comparisons *Comparison of* the *Maniola jurtina genome with Heliconious melpomene and Bicyclus anynana*. The dot plots were generated using Mummer. The plots show relatedness of *M. jurtina* with (A) *H. melpomene* and (B) *B. anynana*. Both of these genomes were taken as references (x-axis) and queried using *M. jurtina* (y-axis) genome. In both plots, blue, green and orange colored dots represent the unique forward, unique reverse and repetitive alignments respectively. Plot B shows more consistent and contiguous alignments than plot A. The dot plots were generated using https://dnanexus.github.io/dot/.

The phylogenetic analysis showed that *M. jurtina* is more closely related to *B. anynana* than to *H. melpomene* or *M. cinxia*. The divergence time between *M. jurtina* and *B. anynana* was estimated to be around 34 MYA and that between *M. jurtina* and *H. melpomene* is estimated as 57 MYA (Figure 3B and see Table S9 for divergence time calibrations). Whole genome alignments, using Mummer -v3.1.0 (Kurtz *et al.* 2004) between *M. jurtina* – *B. anynana* and *M. jurtina* - *H. melpomene* were also performed to confirm this relatedness (Figure 4). In the dated phylogeny, the most species rich family Nymphalidae has remained stable and diverged from Papilionidae around 90 MY ago. This age is also supported by previously published butterfly phylogenies (Wahlberg *et al.* 2013; Espeland *et al.* 2018).

### Analysis of gene family evolution

CAFE models the evolution of gene family size across a species phylogeny under a ML birth–death model of gene gain and loss and simultaneously reconstructs ML ancestral gene family sizes for all internal nodes, allowing the detection of expanded gene families within lineages. We ran CAFE on our matrix of gene family sizes generated by OrthoFinder and modeled their evolution along the dated species tree. Genes involved in binding, metabolism and transport of natural or synthetic allelochemicals are particularly found to be rapidly evolving in *M. jurtina* (Figure 3B).

### Analysis of spot pattern related genes

Dowdeswell, Fisher and Ford first studied the island-specific wing-spot patterns in *M. jurtina* on the isles of Scilly (Dowdeswell *et al.* 1949), and this work was continued for more than 20 years (reviewed in (Ford 1965)). Their major findings, which became a cornerstone of ecological genetics, have been re-visited and largely re-confirmed with contemporary data (Baxter *et al.* 2017). Patterns of wing-spot polymorphism have remained unchanged on some islands over 60 years and there is some evidence of genetic differentiation across the Scillies (Baxter *et al.* 2017). Nonetheless, much remains to be done to better understand the underlying genetics of spot pattern variation in this species.

Butterfly wing patterns have long been suggested to be polygenic (Beldade and Brakefield 2002) and recent evidence from *B. anynana* (very closely related to *M. jurtina*) has confirmed this to be the case and strongly suggested that 10-11 different genomic regions may be involved in eye-spot number variation (Rivera-Colón *et al.* 2018 *preprint*) and see (Monteiro and Prudic 2010).

Protein to genome matches were found for 20 out of the 30 candidate genes (Table 3). We further cross checked this by creating a blast database of the 1347 homolog spot pattern related proteins from Lepidoptera and then searching the homologs within the *M. jurtina* proteome for matches. This resulted in over 1500 matches (see Table S10).

**Table 3:** Candidate wing spot patterning genes obtained from a literature search are listed in column 1. Column 2 has the number of annotated orthologs across Lepidoptera in our NCBI protein database search using the full gene name as listed and alternate names (comma separated). Column 3 presents the number of proteins per gene that matched in our Exonerate workflow

	Candidate gene *(alt. name in brackets) [Reference]*	NCBI Protein Matches within Lepidoptera *(n)*	protein2genome matches against the *Maniola* genome *(n)*
1	*Ap (**Beldade et al. 2005**)*	1	0
2	*APC (**Saenko et al. 2010**)*	18	4
3	*Apterous / apterousA (apA) (**Beldade et al. 2005*; *Prakash and Monteiro 2018**)*	25	4
4	*Black (**Walker and Monteiro 2013**)*	5	0
5	*C2 domain-containing protein 5 (C2CD5) (**Rivera-Colón et al. 2018 preprint**)*	32	7
6	*Calcium-activated potassium channel slowpoke (slo) (**Özsu and Monteiro 2017*; *Rivera-Colón et al. 2018 preprint**)*	205	191
7	*Cortex (**Nadeau et al. 2016*; *Callier 2018**)*	26	0
8	*Decapentaplegic (dpp) (**Monteiro et al. 2013*; *Connahs et al. 2017 preprint**)*	145	86
9	*Distal-less (Dll) (**Koch et al. 2003*; *Reed and Serfas 2004*; *Monteiro et al. 2013**)*	47	1
10	*Doublesex (dsx) (**Kunte et al. 2014*; *Nishikawa et al. 2015**)*	204	101
11	*DP transcription factor (dp) (**Beldade et al. 2005**)*	4	2
12	*Ecdysone receptor (ecr) (**Koch et al. 1996*; *Koch et al. 2003**)*	101	33
13	*engrailed (en) (**Brunetti et al. 2001**)*	19	0
14	*Geranylgeranyl pyrophosphate synthase (GGPS1) (**Rivera-Colón et al. 2018 preprint**)*	26	10
15	*Hox (**Hombría 2011**)*	39	6
16	*Invected (**Brunetti et al. 2001**)*	15	0
17	*Naked cuticle (**Saenko et al. 2010**)*	16	3
18	*Neutral Ceramidase (Cdase) (**Özsu and Monteiro 2017*; *Rivera-Colón et al. 2018 preprint**)*	32	22
19	*Notch (N) (**Reed and Serfas 2004**)*	117	72
20	*numb (**Rivera-Colón et al. 2018 preprint**)*	40	0
21	*Optix (**Reed et al. 2011*; *Callier 2018**)*	5	3
22	*Phosphoinositide 3-kinase adapter protein 1 (PIK3AP1) (**Rivera-Colón et al. 2018 preprint**)*	58	0
23	*poikilomousa / poik (HM00025) (**Nadeau et al. 2015 preprint**)*	2	0
24	*spaetzle (spz) (**Özsu and Monteiro 2017*; *Rivera-Colón et al. 2018 preprint**)*	65	2
25	*spalt (Sal) (**Brunetti et al. 2001**)*	1	1
26	*Transient receptor potential channel pyrexia (pyx) (**Özsu and Monteiro 2017*; *Rivera-Colón et al. 2018 preprint**)*	74	23
27	*Vermilion (**Beldade et al. 2005**)*	2	0
28	*wingless (wg) (**Monteiro et al. 2006**)*	0	—
29	*WntA (**Callier 2018**)*	1	1
30	*Zinc finger CCCH domain-containing protein 10 (ZC3H10) (**Rivera-Colón et al. 2018 preprint**)*	23	1

Specific experiments now need to be undertaken to further test candidate genes and their possible roles in wing-spot polymorphism, and to revisit other findings from Ford and co-workers (reviewed in (Ford 1965)) in the iconic Scillies study system.

### Concluding remarks

Here we present a high-quality draft assembly and annotation of the butterfly *M. jurtina*. The assembly, along with the cross-species comparisons and elements of key spot-pattern genes will offer a core genomic resource for ongoing work in lepidopterans and other arthropods of ecological importance.
